# Trends and determinants of late antenatal care initiation in three East African countries, 2007–2016: A population based cross-sectional analysis

**DOI:** 10.1371/journal.pgph.0000534

**Published:** 2022-08-15

**Authors:** Chenai Mlandu, Zvifadzo Matsena-Zingoni, Eustasius Musenge

**Affiliations:** School of Public Health, University of Witwatersrand, Johannesburg, South Africa; The University of Texas Health Science Center at Houston School of Public Health - San Antonio Campus, UNITED STATES

## Abstract

Early antenatal care is critical for the mother and newborn’s health. Antenatal care is often delayed in Sub-Saharan Africa. The study aims to examine the trends and determinants of late antenatal care initiation in the Democratic Republic of Congo, Kenya, and Tanzania from 2007–2016. The study employed Demographic Health Surveys data of reproductive-age women seeking antenatal care in the Democratic Republic of Congo (2007-2013/14), Kenya (2008–2014), and Tanzania (2010-2015/16). Bivariate and multivariate analysis was conducted per survey, taking sampling weights into account. The determinants of late antenatal care initiation were measured using multivariate logistic regression models and the trends were assessed using prediction scores. Late antenatal care initiation declined in Tanzania (60.9%-49.8%) and Kenya (67.8%-60.5%) but increased in the Democratic Republic of Congo (56.8%-61.0%) between surveys. In the Democratic Republic of Congo, higher birth order was associated with antenatal care initiation delays from 2007–2014, whilst rural residency (AOR:1.28;95%CI:1.09–1.52), lower maternal education (AOR:1.29;95%CI:1.13–1.47) and lower-income households (AOR:1.30;95%CI:1.08–1.55) were linked to antenatal care initiation delays in 2014. In Kenya, lower maternal education and lower-income households were associated with antenatal care initiation delays from 2008–2014, whilst rural residency (AOR:1.24;95%CI:1.11–1.38) and increased birth order (AOR:1.12; 95%CI:1.01–1.28) were linked to antenatal care initiation delays in 2014. In Tanzania, higher birth order and larger households were linked to antenatal care initiation delays from 2010–2016, whilst antenatal care initiation delays were associated with lower maternal education (OR:1.51;95%CI:1.16–1.97) in 2010 and lower-income households (OR:1.45;95%CI:1.20–1.72) in 2016. Except for the Democratic Republic of Congo, the sub-region is making progress in reducing antenatal care delays. Women from various geographic, educational, parity, and economic groups exhibited varying levels of delayed antenatal care uptake. Increasing women’s access to information platforms and strengthening initiatives that enhance female education, household incomes, and localise services may enhance early antenatal care utilisation.

## Introduction

Maternal mortality reduction remains a top priority in the new Sustainable Development Goals (SDG) 3.1 [1]. Maternal mortality, on the other hand, remains a global issue, with 275,288 deaths in 2015 attributed to pregnancy and related complications [2]. Sub-Saharan Africa (SSA) has the highest regional maternal mortality rate in the world, accounting for 66% (201 000) of global mortality (303 000) in 2015 [2]. Furthermore, countries in the East African sub-region including the Democratic Republic of the Congo (DRC), Kenya, and Tanzania were among the ten countries in the world with significant contributions to global mortality in 2015 [2]. However, the vast majority of maternal deaths are avoidable, detectable, and treatable [3, 4].

Antenatal care (ANC) is one of the key strategies for reducing maternal mortality, both directly by detecting and treating pregnancy-related complications and indirectly by identifying women at high risk of birth issues [3, 4]. To lower the risk of maternal death, ANC providers give appropriate medical and educational measures [3–5]. Pregnant women should begin ANC during their first trimester, no later than 16 weeks of pregnancy, according to the World Health Organization (WHO) [5]. Early ANC has also been demonstrated to reduce negative perinatal outcomes such as preterm birth, stillbirth, and low birth weight [6, 7].

Early ANC registration aids health providers in providing timely information and medicines based on the health of the expecting mother [5, 8]. During the first ANC visit, health issues such as the human immunodeficiency virus (HIV) and syphilis are screened, and early discovery of these conditions enhances the health and survival of the unborn child [5, 8]. Furthermore, uptake of therapies such as iron supplementation and immunisations can be life-saving for both mothers and newborn babies if initiated at the early stages of pregnancy [3, 4]. Those who arrive late for ANC miss out on vital health information and interventions [3–5].

Although the World Health Organization (WHO) recommends that the first ANC visit should occur within the first 16 weeks of pregnancy [3], research undertaken in SSA countries has indicated poor early ANC uptake [4–7]. Low early ANC uptake has been linked to characteristics such as rural residency, lack of education and lack of information about ANC, unplanned pregnancy, low income, travel expenses, multiparity, and unemployment [4–7].

Recent studies in the DRC, Kenya, and Tanzania have also found poor early ANC uptake, although the trends in late ANC uptake have not been thoroughly investigated [7]. It is critical to understand how pregnant women’s late care-seeking behavior has changed over time to guide strategic policy actions aimed at improving timely ANC commencement in the SDG era. Thus, this study aims to assess the trends and associated determinants of late ANC initiation in three East African countries: the DRC, Kenya, and Tanzania, from 2007 to 2016.

## Materials and methods

### Study design and setting

The study employs a cross-sectional design with secondary data from publicly available Demographic Health Survey (DHS) surveys in three East African countries, namely the DRC, Kenya, and Tanzania [8, 9].

### Inclusion and exclusion criteria

The study population consisted of DRC, Kenyan, and Tanzanian women aged 15 to 49 years, who had a live birth in the five years preceding each DHS survey between 2007 and 2016. Women who attended at least one ANC visit were included, and only information pertaining to the most recent pregnancy was used.

### Data source and sampling

The DHS Program conducts a nationally representative standardized cross-sectional survey involving women aged 15–49 years [10]. The DHS data is collected through a multistage sampling design, where the first stage involves the selection of enumeration areas (EAs) or clusters drawn from census files and the second stage involves the random selection of individual households within each selected EA or cluster. The probability of selecting each household differs from cluster to cluster; hence, sampling weights were considered in the analysis to account for under and oversampling and to restore the sample’s representativeness [10]. Two most recent rounds of DHS surveys in the three countries between 2007–2016 were used. The latest DHS survey in each country was used as the comparative survey and the preceding survey as the baseline. A total sample size of 42,719 reproductive-age women with a live birth in the five years preceding each DHS survey was used in the analysis in the three countries. More information is provided in S1 Table.

### Study variables

The outcome variable is late ANC initiation categorised as late if the timing of ANC was >4months and early if otherwise [11]. The independent variables were chosen for analysis based on research in SSA [3, 9–11] and availability in DHS surveys, and they represented proxy measures for demographic and socioeconomic variables collected at the time when the woman was pregnant. These variables included the place of residence (rural, urban), mother’s current age group (young women aged 15–24, older women aged 25–49 years), mother’s level of education (primary and no education, secondary and tertiary education), and household wealth status, which was re-categorised as tertiles (poor, middle and rich) in this study, using the household wealth index variable (poorest, poor, middle, richer and richest) contained in the DHS surveys data [12].

### Data analysis

The data were managed, cleaned, coded, and analysed using STATA version 17 (StataCorp, College Station, Texas 77845 USA [13]. The chi-square test was conducted to compare respondents’ demographic and socioeconomic characteristics by late initiation of ANC. The multivariate logistic regression was used to determine the determinants of late ANC initiation. The trends of late ANC initiation over time were first described using proportions plotted on maps using the ArcGIS software [14] and then described using prediction scores (prediction probabilities), estimated after running the multivariate logistic regressions. The differences in the prediction scores between the baseline and latest surveys in each country were assessed using an independent T-test. Similar, independent variables were considered in the multivariate logistic regressions for easy comparison between countries. Significance was set at a p-value of less than 5%.

## Results

### Descriptive analysis

#### Trends of late ANC initiation using proportions and prediction scores

**Fig 1:**
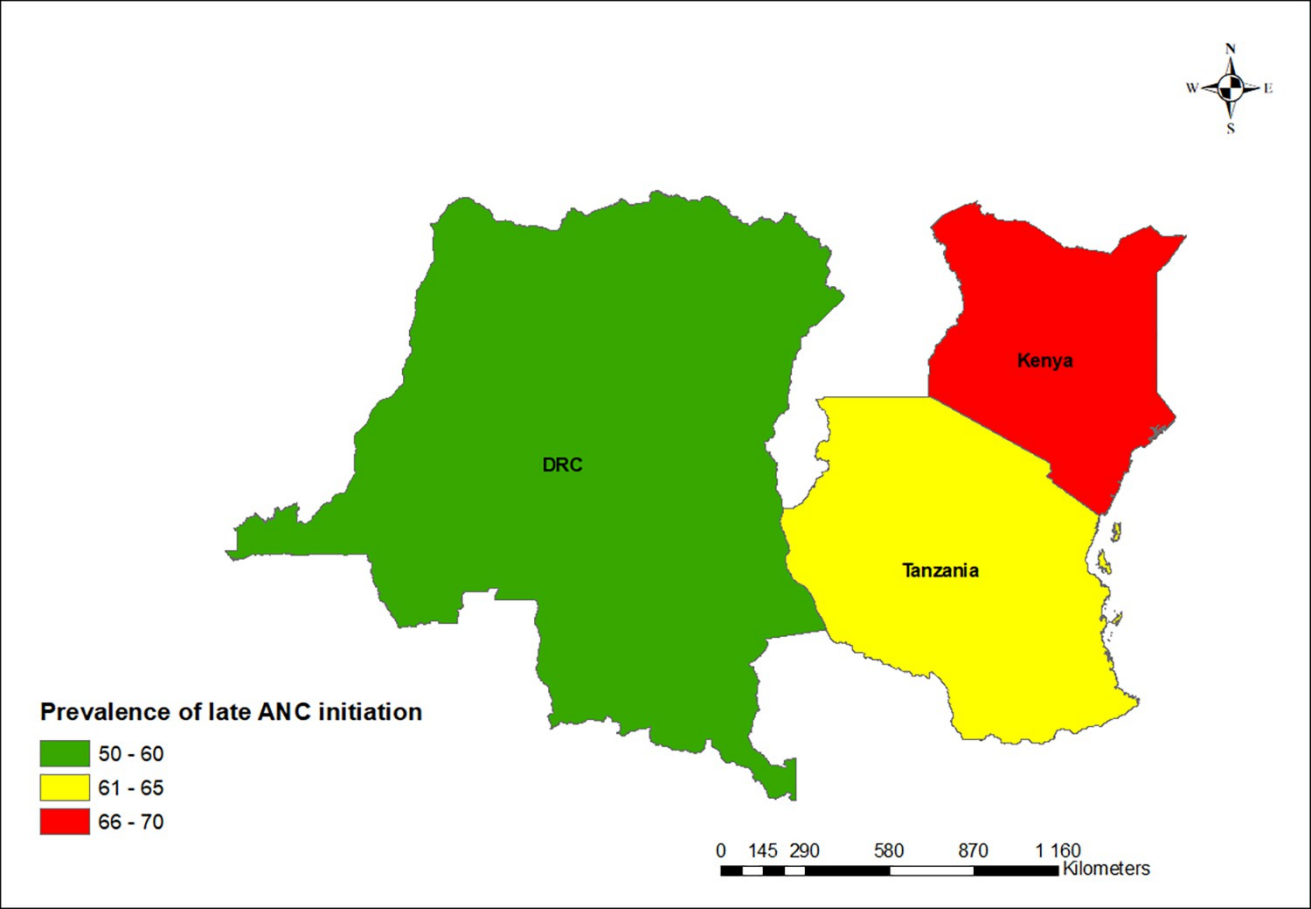
Map showing the prevalence of late ANC initiation in the DRC, Kenya, and Tanzania in the first survey in this study: Base map source at https://spatialdata.dhsprogram.com.

**Fig 2:**
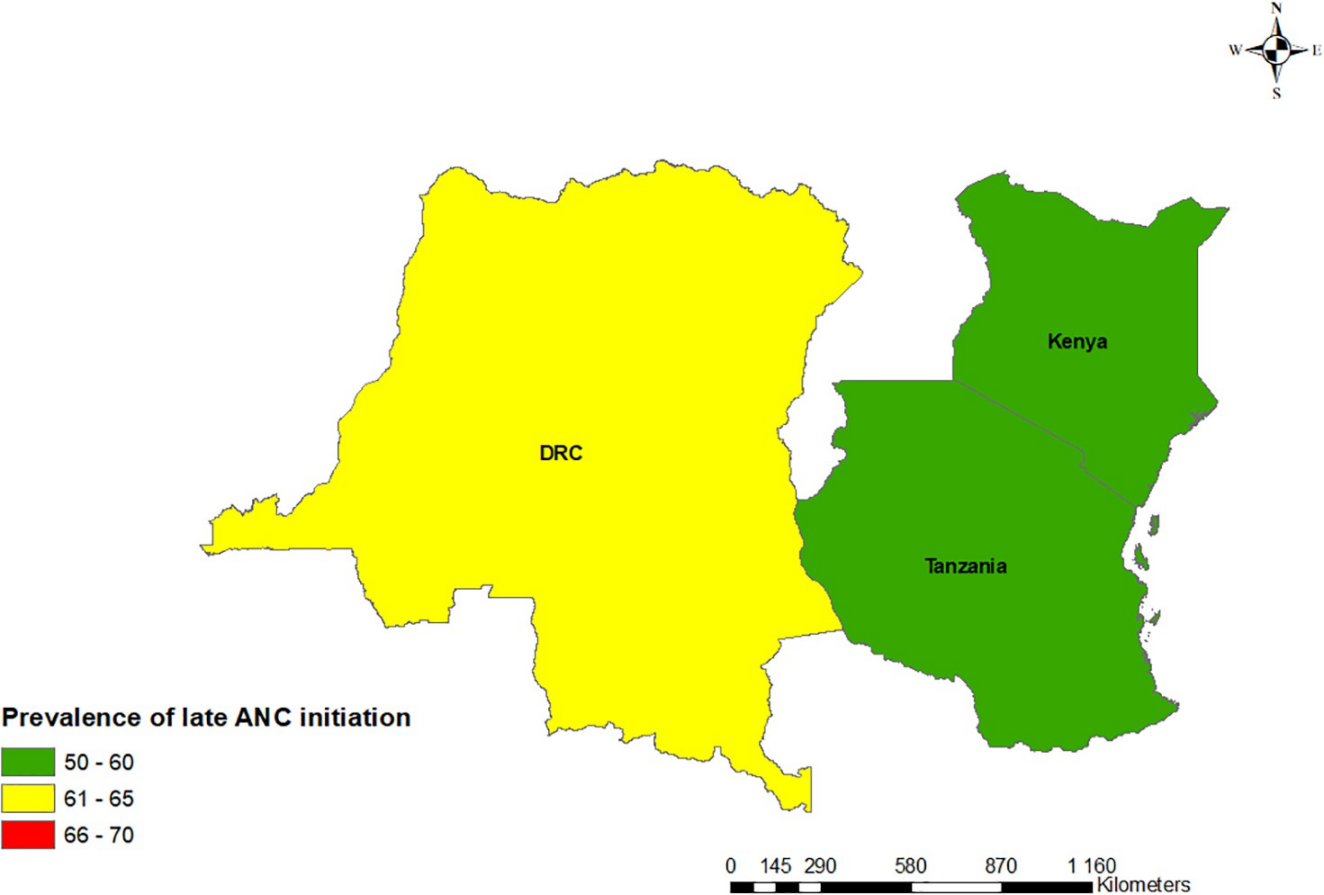
Map showing the prevalence of late ANC initiation in the DRC, Kenya, and Tanzania second survey in this study. Base map source at https://spatialdata.dhsprogram.com.

Using proportions, the results on the map showed a declining trend in the prevalence of late ANC initiation in Kenya and Tanzania between the first and second surveys while the DRC showed an increasing trend as shown in Figs 1 and 2.

**Fig 3:**
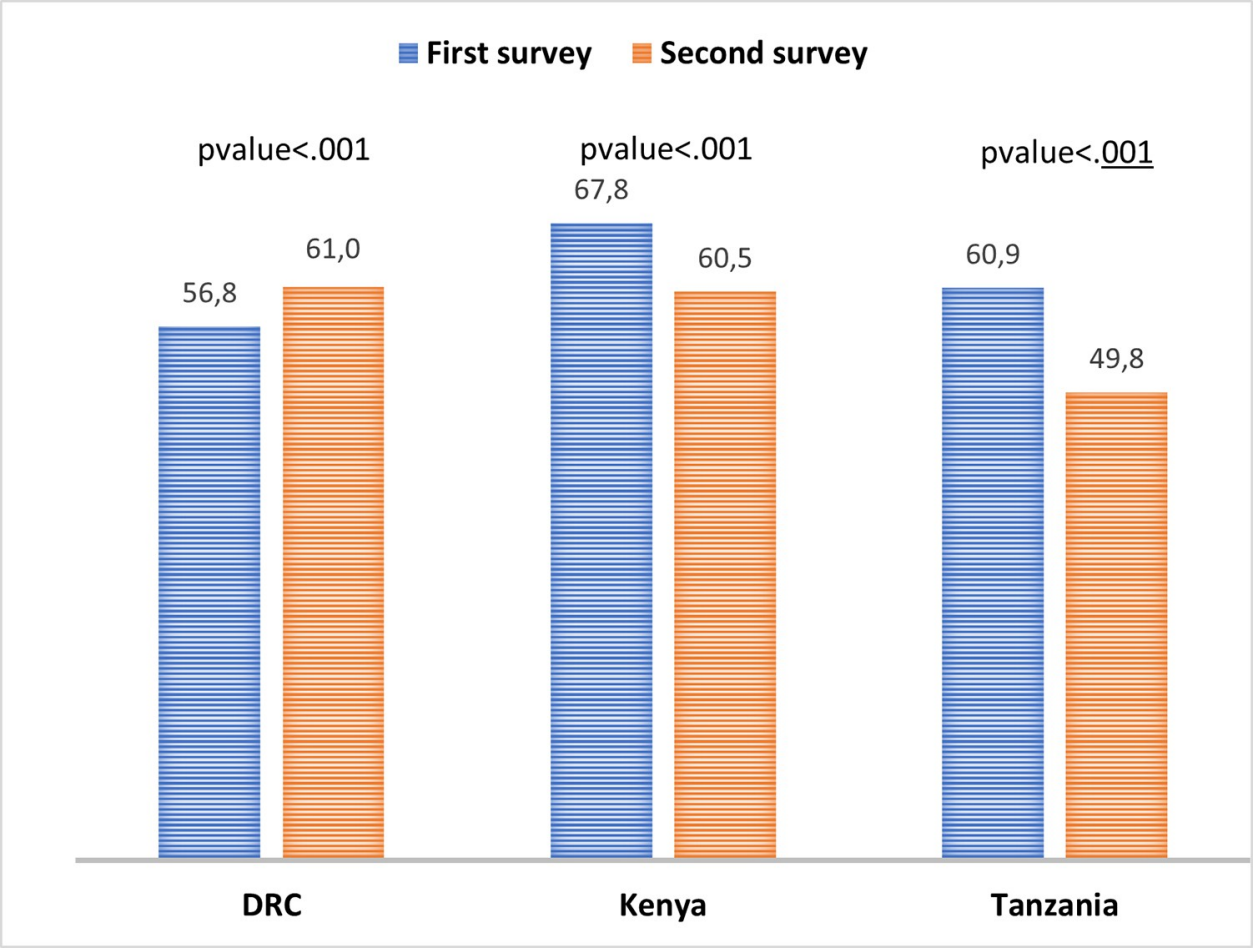
Trends of late ANC initiation in the DRC, Kenya, and Tanzania between the first and second surveys.

Using prediction scores, the trends revealed that Tanzania had the greatest reduction in late ANC initiation, dropping from 60.9% to 49.8% between 2010 and 2016, followed by Kenya, which dropped from 67.8% to 60.5% between 2008 and 2014. Between 2007 and 2014, the trends showed that the DRC had an increase in late ANC initiation, rising from 56.8% to 61.0% (Fig 3).

#### Late ANC initiation by background characteristics of respondents in the combined countries

Table 1 shows late initiation of ANC based on the demographic and socioeconomic characteristics of respondents in the combined countries’ data from the first and second surveys. A total of 13,129 and 29,950 women of reproductive age who had a live birth in the five years preceding each survey were included in this study, in the first and second surveys respectively.

**Table 1:** Late ANC initiation by background characteristics in the combined countries.

	Combined Countries: Late ANC initiation	
**Background characteristics**	First survey (n = 8,242) weighted n(%)	Second survey (n = 16,930) weighted n(%)
**Place of residence**		
Urban	2,241(56.7)	5,336(49.8)
Rural	6,001(63.5)	11,594(61.4)
	(< .001) **	(< .001) **
**Age-group**		
Young women (15–24 years)	2,645(59.9)	5,131(56.4)
Older women (25–49 years)	5,597(62.3)	11,799(57.6)
	(.053)	(.315)
**Education**		
Primary and below	6,468(63.5)	11,812(60.7)
Secondary and above	1,774(55.2)	5,118(50.6)
	(< .001) **	(< .001) **
**Birth order**		
One	4,288(57.7)	9,699(53.7)
Two	3,233(65.2)	5,991(61.9)
Three or more	721(70.3)	1,231(67.1)
	(< .001) **	(< .001) **
**Household size**		
4 or less	2,226(57.6)	5,015(51.3)
5 or more	6,016(63.0)	11,915(60.1)
	(< .001)**	(< .001)**
**Household wealth**		
Poor	3,242(64.2)	7,399(63.8)
Middle	1,801(64.6)	3,408(59.9)
Rich	3,016(57.1)	6,113(49.7)
	(< .001)**	(< .001)**

In the first survey, rural women initiated ANC later (63.5%) than urban women (56.7%), and a similar pattern was observed between rural women (61.4%) and urban women (49.8%) in the second survey. In the first survey, older women aged 25–49 years started ANC later (62.3%) than younger women aged 15–24 years (59.9%). In the second survey, a similar pattern was observed between older women (57.6%) and younger women (56.4%), but the differences between the two age groups were not statistically significant in either survey. In the first survey, women with primary or no education delayed their first ANC visit (63.5%) more than women with secondary or tertiary education (55.2%). In the second survey, the same pattern was observed among women with primary or no education (60.7%) and women with secondary or tertiary education (50.6%)

In the first survey, women with birth order of three or more started ANC later (70.3%) than those with birth order of two (65.2%) or one (57.7%). In the second survey, women with birth order of three or more also initiated ANC later (67.1%) than those birth order of two (61.9%) or one (53.7%). Women in households with 5 or more members started ANC later (63.0%) than women in households with 4 or fewer members (57.6%) in the first survey. In the second survey, women in households with 5 or more members (60.1%) and women in households with 4 or fewer members (51.3%) showed a similar pattern. In the first survey, women in poor-income households delayed their first ANC visit (64.2%%) more than women in rich-income (57.1%) households. In the second survey, women in poor-income households also waited longer to start ANC (63.8%) than women in middle-income (59.9%) or rich-income (49.7%) households.

### Late ANC initiation by background characteristics by country

Table 2 shows late initiation of ANC by demographic and socioeconomic characteristics of respondents for the various countries, i.e., DRC, Kenya, and Tanzania, in the first and second surveys. In the DRC, a total of 4,559 and 8,941 reproductive-age women with a live birth in the five years preceding each survey were included in this study in 2007 (baseline survey) and 2014 (comparative survey) respectively. In 2007, rural women started ANC later (58.7%) than urban women (54.7%), and in 2014, a similar pattern was found among rural women (64.9%) and urban women (52.5%). In 2007, however, the differences between rural and urban areas were not statistically significant.

**Table 2:** Late ANC initiation by background characteristics in the DRC, Kenya and Tanzania.

	DRC: Late ANC initiation		Kenya: Late ANC initiation		Tanzania: Late ANC initiation	
**Background characteristics**	2007(n = 2,621) weighted n(%)	2014(n = 5,443) weighted n(%)	2008(n = 2,412) weighted n(%)	2014(n = 8,067) weighted n(%)	2010(n = 3,209) weighted n(%)	2016(n = 3,410) weighted n(%)
**Place of residence**						
Urban	1,067(54.7)	1,687(52.5)	488(62.6)	2,780(51.4)	686(56.0)	870(41.7)
Rural	1,553(58.7)	3,757(64.9)	1,924(69.2)	5,287(63.8)	2,523(62.7)	2,541(53.0)
	(.233)	(< .001)**	(.018)**	(.001)**	(.013)**	(< .001)**
**Age-group**						
Younger women (15–24 years)	844(53.7)	1,624(58.9)	836(69.2)	2,422(59.1)	965(58.8)	1,085(48.2)
Older women (25–49 years)	1,777(58.7)	3,819(61.2)	1,576(67.0)	5,645(58.8)	2,244(62.2)	2,326(50.2)
	(.024)**	(.112)	(.319)	(.802)	(.094)	(.236)
**Education**						
Primary and below	1,640(59.1)	3,324(65.3)	1,810(70.5)	5,522(64.1)	3,018(62.3)	2,956(51.8)
Secondary and above	981(53.8)	2,119(54.1)	602(60.7)	2,545(50.1)	191(47.7)	454(40.3)
	(.031)**	(< .001)**	(.002)**	(< .001)**	(< .001)**	(< .001)**
**Birth order**						
One	1,157(52.6)	2,381(56.9)	1,413(66.8)	5,383(56.6)	1,718(56.1)	1,935(44.2)
Two	1,139(59.8)	2,399(62.5)	840(71.4)	2,323(63.5)	1,254(66.7)	1,268(58.3)
Three or more	325(65.5)	663(68.3)	159(72.4)	360(67.0)	237(76.8)	207(63.8)
	(< .001)**	(< .001)**	(.035)**	(< .001)**	(< .001)**	(< .001)**
**Household size**						
4 or less	609(54.9)	1,258(57.9)	844(64.5)	3,033(53.2)	773(53.5)	724(38.2)
5 or more	2,012(57.6)	4,186(61.3)	1,568(69.7)	5,033(62.9)	2,436(64.1)	2,686(53.9)
	(.367)	(.025)**	(.015)**	(< .001)**	(< .001)**	(< .001)**
**Household wealth**						
Poor	1,033(57.3)	2,331(66.0)	993(72.6)	3,455(66.2)	1,398(64.6)	1,613(56.8)
Middle	620(62.0)	1,161(63.7)	478(70.8)	1,583(61.9)	703(63.2)	663(50.4)
Rich	967(53.8)	1,951(53.5)	941(62.1)	3,029(51.1)	1,108(56.2)	1,134(41.6)
	(.100)	(< .001)**	(< .001)**	(< .001)**	(< .001)**	(< .001)**

In 2007, older women aged 25–49 years commenced ANC later (58.7%) than younger women aged 15–24 years (53.7%). In 2014, older women (61.2%) and younger women (58.9%) followed a similar pattern. However, the differences between older and younger age groups were only statistically significant in 2007. In 2007, women with primary or no education started ANC later (59.1%) than women with secondary or tertiary education (53.8%). A similar pattern was observed among women with primary or no education (65.3%) and women with secondary or tertiary education (54.2%) in 2014.

In 2007, women with birth order of three or more began ANC later (65.5%) than those with birth order of two (59.8%) or one (52.6%). In 2014, women with birth order of three or more also commenced ANC later (68.3%) than those with birth order of two (62.5%) or one (56.9%). In 2007, higher proportions of women in households with five or more members delayed their first ANC visit (57.6%) than women in households with four or fewer members (54.9%). In 2014, a similar pattern was observed for women in households with five or more members (61.3%) and women in households with four or fewer members (57.9%). In 2007, lower proportions of women in low-income families started ANC later (57.3%) than women in rich-income families (53.8%). In 2014, women in low-income families delayed ANC initiation (66.0%) more than women in middle-income (63.7%) or rich-income families (53.5%)

In Kenya, a total of 3,512 and 13,766 reproductive-age women with a live birth in the five years preceding each survey were included in this study in 2008 (baseline survey) and 2014 (comparative survey) respectively. Women in rural areas started ANC later (69.2%) than women in urban areas (62.6%) in 2008. In 2014, a similar pattern was observed among rural women (63.8%) and urban women (51.4%). In 2008, younger women aged 15–24 years commenced ANC later (69.2%) than older women aged 25–49 years (67.0%). Younger women also started ANC later (59.1%) than older women (58.8%) in 2014. However, for both surveys, the differences in younger and older age groups were not statistically significant.

In 2008, women with primary or no education started ANC later (70.5%) than women with secondary or tertiary education (60.7%). In 2014, women with primary or no education also started ANC later (64.1%) than women with secondary or tertiary education (50.1%). In 2008, mothers with birth order of three or more initiated ANC later (72.4%) than those with birth order of two (71.4%) or one (66.8%). In 2014, mothers with birth order of three or more (67.0%) also started ANC later than those with birth order of two (63.5%) or one (56.6%).

In 2008, women in families with five or more members delayed beginning ANC (69.7%) more than women in households with four or fewer members (64.5%). In 2014, a similar tendency was observed among women in families with five or more members (62.9%) and women in families with four or fewer members (53.2%). In 2008, women in poor-income households started ANC later (72.6%) than women in middle-income (70.8%) or rich-income (62.1%) households. In 2014, women in poor-income households also began ANC later (66.2%) than women in middle-income (61.9%) or rich-income households (51.1%).

In Tanzania, a total of 5,058 and 6,873 reproductive-age women were included in this study in 2010 (baseline survey) and 2016 (comparative survey) respectively. In 2010, rural women delayed their first ANC visit (62.7%) more than urban women (56.0%). In 2016, rural women also started ANC later (53.0%) than urban women (41.7%). In 2010, older women aged 25–49 years began ANC later (62,2%) than younger women aged 15–24 years (58.8%). In 2016, older women aged 25–49 years also started ANC later (50.2%) than younger women aged 15–24 years (48.2%). In both surveys, however, differences between older and younger age groups were not statistically significant.

In 2010, women with primary or no education delayed ANC initiation (62.3%) more than those with secondary or tertiary education (47.7%). In 2016, a similar pattern was observed among women with primary or no education (51.8%) and women with secondary or tertiary education (40.3%). In 2010, mothers with birth order of three or more attended their first ANC visit later (76.8%) than those with birth order of two (66.7%) or one (56.1%). Similarly, in 2016, mothers with birth order of three or more began ANC later (63.8%) than those with birth order of two (58.3%) or one (44.2%).

In 2010, women in families with five or more members began ANC later (64.1%) than women in families with four or fewer members (53,5%). In 2016, women in families with five or more members also commenced ANC later (53.9%) than women in households with four or fewer members (38.2%). In 2010, women in poor-income households delayed their first ANC visit (64.6%) more than women in middle-income (63.2%) or rich-income households (56.2%). In 2016, women in poor-income households also started their first ANC visit (56.8%) more than women in middle-income (50.4%) or rich-income (41.6%) households.

#### Determinants of late ANC initiation in the combined countries

Table 3 shows the determinants of late ANC initiation in the combined countries’ data in the first and second surveys, adjusted for the country of residence. Late ANC initiation was associated with the mother’s education, birth order, household size, and household wealth in the first and second surveys, and the location of residence was linked to late ANC initiation in the second survey.

**Table 3:** Determinants of late ANC initiation in the combined countries.

	Combined Countries: Late ANC initiation	
	First Survey	Second Survey
	AOR(95%CI)	AOR(95%CI)
**Variables**		
**Country**		
DRC	**Reference**	**Reference**
Kenya	1.70(1.48–1.96)**	1.04(0.96–1.12)
Tanzania	1.16(1.03–1.31)**	0.61(0.56–0.66)**
**Place of residence**		
Urban	**Reference**	**Reference**
Rural	0.97(0.84–1.11)	1.21(1.12–1.31)**
**Age-group**		
Younger women (15–24 years)	**Reference**	**Reference**
Older women (25–49 years)	1.04(0.94–1.16)	0.96(0.90–1.03)
**Education**		
Secondary and above	**Reference**	**Reference**
Primary and below	1.26(1.10–1.44)**	1.33(1.23–1.43)**
**Birth order**		
One	**Reference**	**Reference**
Two	1.33(1.20–1.47)**	1.22(1.14–1.31)**
Three or more	1.68(1.38–2.06)**	1.45(1.26–1.66)**
**Household size**		
4 or less	**Reference**	**Reference**
5 or more	1.21(1.08–1.34)**	1.31(1.22–1.40)**
**Household wealth**		
Rich	**Reference**	**Reference**
Middle	1.29(1.10–1.50)**	1.21(1.10–1.33)**
Poor	1.21(1.06–1.39)**	1.34(1.23–1.46)**

The results showed that the odds of late ANC initiation were significantly higher among women with primary or no education in the first survey (AOR:1.26; 95%CI:1.10–1.44) and second survey (AOR:1.33;95%CI:1.23–1.43). It was also observed that mothers with birth order of two (AOR:1.33;95%CI:1.20–1.47) and those with birth order of three or more (AOR:1.68;95%CI:1.38–2.06) had significantly greater odds of starting ANC late in the first survey. A similar observation was made in the second survey, mothers with birth order of two (AOR:1.22;95%CI:1.14–1.31) and those with birth order of three or more (AOR:1.45;95%CI:1.26–1.66) had significantly higher odds of waiting longer to start ANC.

The results also showed that the odds of delayed ANC initiation were significantly higher among women living in households with five or more members in the first survey (AOR:1.21:95%CI:1.08–1.34) and second survey (AOR:1.31;95%CI:1.22–1.40). The study also established that women from poor-income (AOR:1.21;95%CI:1.06–1.39) and middle-income (AOR:1.29;95%CI:1.10–1.50) households had significantly greater odds of initiating ANC late in the first survey. These findings were also observed in the second survey, the odds of delaying ANC initiation were significantly higher among women from poor-income (AOR:1.34;95%CI:1.23–1.46) and middle-income (AOR:1.21;95%CI:1.10–1.33) households. The findings also revealed that women residing in rural areas had significantly greater odds of late ANC initiation (AOR:1.21;95%CI:1.12–1.31) in the second survey.

#### Determinants of late ANC initiation in the individual countries

Table 4 shows the determinants of late ANC initiation in the DRC, Kenya and Tanzania by survey. In the DRC, birth order was associated with late ANC initiation in 2007 and 2014, and the place of residence, mother’s education, and household wealth were associated with late ANC initiation in 2014. The results showed that the odds of initiating ANC late significantly increased by 31% (95%CI:1.09–1.57) for women with birth order of two, and by 65% (95%CI:1.22–2.24) for women with birth order of three or more in 2007. Similarly, in 2014, women with birth order of two (AOR:1.16;95% CI:1.03–1.32) and women with birth order of three or more (AOR:1.51;95% CI:1.22–1.87) had significantly greater odds of late ANC initiation.

**Table 4:** Determinants of late ANC initiation in the DRC, Kenya and Tanzania.

	DRC: Late ANC initiation		Kenya: Late ANC initiation		Tanzania: Late ANC initiation	
	Survey (2007)	Survey (2014)	Survey (2008)	Survey(2014)	Survey(2010)	Survey(2016)
**Variables**	AOR(95%CI)	AOR(95%CI)	AOR(95%CI)	AOR(95%CI)	AOR(95%CI)	AOR(95%CI)
**Place of residence**						
Urban	**Reference**	**Reference**	**Reference**	**Reference**	**Reference**	**Reference**
Rural	1.03(0.81–1.32)	1.28(1.09–1.52)**	0.93(0.69–1.25)	1.24(1.11–1.38)**	0.96(0.78–1.19)	1.06(0.90–1.25)
**Age-group**						
Younger women (15–24 years)	**Reference**	**Reference**	**Reference**	**Reference**	**Reference**	**Reference**
Adult women (25–49 years)	1.17(0.97–1.42)	1.00(0.87–1.15)	0.89(0.72–1.11)	0.94(0.85–1.05)	1.05(0.90–1.23)	0.94(0.83–1.07)
**Education**						
Secondary and above	**Reference**	**Reference**	**Reference**	**Reference**	**Reference**	**Reference**
Primary and below	1.16(0.95–1.42)	1.29(1.13–1.47)**	1.33(1.04–1.70)**	1.42(1.28–1.59)**	1.51(1.16–1.97)**	1.18(0.99–1.40)
**Birth order**						
One	**Reference**	**Reference**	**Reference**	**Reference**	**Reference**	**Reference**
Two	1.31(1.09–1.57)**	1.16(1.03–1.32)**	1.18(0.95–1.47)	1.12(1.00–1.24)**	1.43(1.23–1.66)**	1.53(1.34–1.74)**
Three or more	1.65(1.22–2.24)**	1.51(1.22–1.87)**	1.10(0.71–1.71)	1.17(0.93–1.47)	2.20(1.58–3.06)**	1.76(1.35–2.30)**
**Household size**						
4 or less	**Reference**	**Reference**	**Reference**	**Reference**	**Reference**	**Reference**
5 or more	1.01(0.81–1.24)	1.16(1.00–1.34)	1.15(0.92–1.42)	1.26(1.14–1.39)**	1.38(1.18–1.62)**	1.65(1.44–1.90)**
**Household wealth**						
Rich	**Reference**	**Reference**	**Reference**	**Reference**	**Reference**	**Reference**
Middle	1.32(1.00–1.76)	1.21(0.99–1.48)	1.38(1.01–1.88)**	1.22(1.06–1.39)**	1.14(0.92–1.41)	1.19(0.99–1.44)
Poor	1.04(0.80–1.35)	1.30(1.08–1.55)**	1.42(1.07–1.88)**	1.29(1.15–1.46)**	1.21(1.00–1.46)	1.45(1.23–1.72)**

According to the study, women residing in rural areas had significantly increased odds of initiating ANC late (AOR:1.28;95%CI:1.09–1.52) in 2014. It was also established that women with primary or no education had significantly higher odds of late ANC initiation (AOR:1.29:95%CI:1.13–1.47) in 2014. The findings also revealed that women living in poor-income households had significantly increased odds of late ANC initiation (AOR:1.30; 95%CI:1.08–1.55) in 2014.

In Kenya, the mother’s education and household wealth were associated with late ANC initiation in 2008 and 2014, and the place of residence, birth order, and household size were linked to late ANC initiation in 2014. The results showed that women with primary or no education had significantly higher odds of late ANC initiation (AOR:1.33;95%CI:1.04–1.70) in 2008 and (AOR:1.42;95%CI:1.28–1.59) in 2014. The findings also showed that women in poor-income (AOR:1.42;95%CI:1.07–1.88) and middle-income (AOR:1.38; 95% CI:1.01–1.88) households had significantly higher odds of late ANC initiation in 2008. These findings were also similar in 2014, the odds of late ANC initiation were significantly higher among women in poor-income (AOR:1.29;95%CI:1.15–1.46) and middle-income (AOR:1.22;95%CI:1.06–1.39) households.

The findings also revealed that rural women had significantly higher odds of late ANC initiation (AOR:1.24;95%CI:1.11–1.38) in 2014. The study also found that women with birth order of two had significantly higher odds of delayed ANC initiation (AOR:1.12:95%CI:1.01–1.24) in 2014. Women living in households with 5 or more members also had significantly increased odds of late ANC initiation (AOR:1.38;95%CI:1.18–1.62) in 2014.

In Tanzania, late initiation of ANC was associated with birth order and household size in 2010 and 2016, and delays in ANC initiation were associated with the mother’s education in 2010 and household wealth in 2016. The results showed the odds of initiating ANC late significantly increased among mothers with birth order of two (AOR:1.43;95%CI:1.23–1.66) and those with birth order of three or more (AOR:2.20;95%CI:1.58–3.16). Similarly, in 2016, the odds of starting ANC late significantly increased among mothers with birth order of two (AOR:1.53;95%CI:1.34–1.74) and those with birth order of three or more (AOR:1.76:95% CI:1.35–2.30).

The study also found that the odds of initiating ANC late significantly increased among women residing in households with five or more members (OR: 1.38; 95% CI: 1.18–1.62) in 2010 and (AOR:1.65;95%CI:1.44–1.90) in 2016. The findings also revealed that women with primary or no education had significantly increased odds of starting ANC late (AOR:1.51;95%CI:1.16–1.97) in 2010. The study also showed that women from low-income households had significantly greater odds of late ANC initiation (AOR:1.45;95%CI:1.23–1.72) in 2016.

## Discussion

The present study attempts to examine the trends and determinants of late ANC initiation using multiple rounds of DHS surveys from 2007 to 2016 in the DRC, Kenya, and Tanzania. The results of this study indicated that there was a general downward trend of late ANC initiation in Kenya between 2008 and 2014 and Tanzania between 2010 and 2016, however, there was an upward trend in the DRC between 2007 and 2014. The findings indicate uneven progress in the reduction of delays in late initiation of ANC over time within East Africa. Differences in the economic, and social environments, as well as the implementation of diverse maternal health policies, could explain this variation.

In the DRC and Kenya, rural women were more likely to delay ANC. Previous research has shown similar results [7, 15]. Women in rural areas may face access issues and be less aware of health issues than women in urban areas [15, 16]. Rural women may also have additional cultural barriers prohibiting women from starting ANC early, such as seeking the spouse’s approval [17]. Our data also suggested increased barriers to accessing early ANC over time among rural women than urban women in the DRC and Kenya, as rural residence was a risk factor in the current surveys. The urban-rural disparities can be bridged by governments taking strong initiatives to improve access to early ANC among rural women. Among the three countries under study, Tanzania has a long standing health policy focusing on the expansion of health services in rural areas since the 1990s [18]. Such initiatives which put much emphasis on rural development could improve access to maternal healthcare among rural women [18].

Consistent with previous studies, the study found that women with lower household incomes were more likely to delay ANC initiation in the DRC, Kenya, and Tanzania [19, 20]. High costs of care, transportation problems, and poor service provision could be affecting low-income women, resulting in ANC delays [12, 20]. The provision of free maternal services and the expansion of health care facilities could improve early access to ANC for low-income women [21]. Such initiatives have been adopted by the Kenyan and Tanzanian governments [18, 22, 23]. However, despite government efforts, our analysis also showed that low-income women in Kenya and Tanzania continue to face more barriers to accessing early ANC than high-income women, as household wealth was a risk factor in the current surveys. These findings show that governments may need to adopt a holistic approach in addressing barriers to accessing early ANC among low-income women as multiplura of factors may be at play [24, 25].

Our findings also revealed that women with poor educational attainment in the DRC, Kenya, and Tanzania were more likely to commence ANC late. This is corroborated by research conducted in Zambia and Ethiopia, which found that women with poor levels of education were more likely to delay ANC enrollment [16, 24]. Uneducated women may have a limited understanding of ANC services and the benefits of early ANC commencement to safe pregnancy. Early ANC initiation could be improved through community interventions such as mass media campaigns and government policies promoting female education [12, 21]. Our findings also indicated progress in closing differences in late access of ANC among low and high-educated women in Tanzania, as education was only a risk factor in Tanzania in the previous survey. This improvement could be a result of increased maternal literacy rates in Tanzania from 67% in 2004 to 77% in 2016, as indicated by previous reports [12, 25].

The study also revealed that the odds of late ANC initiation increased among mothers with more previous births in the DRC, Kenya, and Tanzania. This was also reported by studies in Cameroon [26] and Uganda [27]. This result could imply that high parity women are less inclined to attend ANC earlier possibly due to previous negative experiences with ANC services [28] and perceive that there is no danger of pregnancy complications because of experience with pregnancy and childbirth [29]. The study also found persistent inequalities in accessing early ANC among women of low-high parity in the DRC and Tanzania and growing inequalities in Kenya, as birth order was a risk factor in the previous and current surveys in the DRC and Kenya, as well as in the second survey in Kenya. These findings emphasise the need to increase community interventions such as mass media campaigns, particularly for multigravida women to improve the timely use of maternal healthcare services among these women.

In Kenya and Tanzania, women with a household size of at least five members were more likely to initiate ANC late, according to the study. The findings are similar to a previous study in Ethiopia [19]. Potential reasons include financial constraints due to the high expense of maintaining a large household on a small income, as well as a preoccupation with family responsibilities that result in neglect of one’s health [19]. Our study also showed persistent differences in access to early ANC in Tanzania between women from large and small households, as well as rising inequalities in Kenya, as household size was a risk factor in the previous and current Tanzanian surveys, and the current Kenyan survey. Increasing community education programs through accessible media platforms and improving the proximity of health services through mobile clinics could break barriers to uptake of early ANC among women bearing responsibilities of larger households [12, 30].

### Strengths and limitations of the study

The strength of this study is that the study used nationally representative data therefore the findings are generalisable and applicable to countrywide policies and interventions. The study also assessed trends and determinants of late ANC initiation across various countries in the East African sub-region. Thus, highlighting the context-specific characteristics in various countries that must be considered when establishing policies and interventions to improve early access to ANC. However, the study has several limitations, including recall bias due to the self-reported nature of the data and data collected for the five preceding years. Our research also depended on the completeness of data on variables of interest in all rounds of the DHS surveys; as a result, the study did not evaluate other variables such as employment status due to insufficient data. Due to the cross-sectional nature of the study, it was also not possible to establish temporal causality

## Conclusion

Countries within East Africa experienced a reduction in late initiation of ANC over time except for the DRC. Rural women, women with low levels of education and socioeconomic status, women with high parity, and women living in large households continue to face challenges in utilising ANC services early. Public health officers should work on increasing awareness about the benefits of early initiation of ANC to all pregnant women. Governments should implement and improve policies that eliminate barriers to accessing maternal healthcare, especially among women of low economic status. These policies should entail making services accessible to women who cannot afford them, increasing access to healthcare for pregnant women who may have to travel long distances, and improving the provision of services at health facilities. Furthermore, government policies aimed at increasing female education would improve the early uptake of ANC services.

## Supporting information

S1 Table DHS survey data used in the study analysis. (DOCX)

## References

[1] UNDP. SUSTAINABLE DEVELOPMENT GOALS 2021 [Available from: https://www.africa.undp.org/content/rba/en/home/sustainable-development-goals.html.

[2] World Health Organization. Trends in maternal mortality: 1990–2015: Estimates from WHO, UNICEF, UNFPA, World Bank Group and the United Nations Population Division: World Health Organization; 2015.

[3] World Health Organisation. WHO recommendations on antenatal care for a positive pregnancy experience: World Health Organization; 2016. 28079998

[4] Wolde HF, Tsegaye AT, Sisay MM. Late initiation of antenatal care and associated factors among pregnant women in Addis Zemen primary hospital, South Gondar, Ethiopia. Reproductive Health. 2019;16(1):1–8. 31151402 10.1186/s12978-019-0745-2 PMC6544982

[5] Ebonwu J, Mumbauer A, Uys M, Wainberg ML, Medina-Marino A. Determinants of late antenatal care presentation in rural and peri-urban communities in South Africa: A cross-sectional study. PLoS One. 2018;13(3):e0191903. doi: 10.1371/journal.pone.0191903 29518082 PMC5843210

[6] Okedo-Alex IN, Akamike IC, Ezeanosike OB, Uneke CJ. Determinants of antenatal care utilisation in sub-Saharan Africa: a systematic review. BMJ Open. 2019;9(10):e031890. doi: 10.1136/bmjopen-2019-031890 31594900 PMC6797296

[7] Alem AZ, Yeshaw Y, Liyew AM, Tesema GA, Alamneh TS, Worku MG, et al. Timely initiation of antenatal care and its associated factors among pregnant women in sub-Saharan Africa: A multicountry analysis of Demographic and Health Surveys. PloS One. 2022;17(1):e0262411. doi: 10.1371/journal.pone.0262411 35007296 PMC8746770

[8] East African Community. The Democratic Republic of the Congo joins EAC as its 7th Member 2022 [Available from: https://www.eac.int/press-releases/2402-the-democratic-republic-of-the-congo-joins-eac-as-its-7th-member.

[9] Ruktanonchai CW, Nilsen K, Alegana VA, Bosco C, Ayiko R, Seven Kajeguka AC, et al. Temporal trends in spatial inequalities of maternal and newborn health services among four east African countries, 1999–2015. BMC Public Health. 2018;18(1):1–13. doi: 10.1186/s12889-018-6241-8 30514269 PMC6278077

[10] USAID. The DHS Program 2022 [cited 2022 7 January]. Available from: https://dhsprogram.com/data/.

[11] Paudel YR, Jha T, Mehata S. Timing of first antenatal care (ANC) and inequalities in early initiation of ANC in Nepal. Frontiers in public health. 2017;5:242. doi: 10.3389/fpubh.2017.00242 28955707 PMC5600995

[12] Rwabilimbo AG, Ahmed KY, Page A, Ogbo FA. Trends and factors associated with the utilisation of antenatal care services during the millennium development goals era in Tanzania. Tropical Medicine and Health. 2020;48(1):1–16. doi: 10.1186/s41182-020-00226-7 32518496 PMC7268642

[13] StataCorp L. Stata Statistical Software: Release 17. College Station, TX: StataCorp LLC; 2019.

[14] ArcGIS Release 10.1 [Internet]. Redlands, CA. 2012.

[15] Tesfaye G, Loxton D, Chojenta C, Semahegn A, Smith R. Delayed initiation of antenatal care and associated factors in Ethiopia: a systematic review and meta-analysis. Reproductive Health. 2017;14(1):1–17. 29141675 10.1186/s12978-017-0412-4 PMC5688656

[16] Teshale AB, Tesema GA. Prevalence and associated factors of delayed first antenatal care booking among reproductive age women in Ethiopia; a multilevel analysis of EDHS 2016 data. PloS One. 2020;15(7):e0235538. doi: 10.1371/journal.pone.0235538 32628700 PMC7337309

[17] Clark J, Sweet L, Nyoni S, Ward PR. Improving male involvement in antenatal care in low and middle-income countries to prevent mother to child transmission of HIV: a realist review. PloS One. 2020;15(10):e0240087. doi: 10.1371/journal.pone.0240087 33057353 PMC7561142

[18] Dominicus DA, Akamatsu T. Health policy and implementations in Tanzania. The Keio journal of medicine. 1989;38(2):192–200. doi: 10.2302/kjm.38.192 2779061

[19] Dewau R, Muche A, Fentaw Z, Yalew M, Bitew G, Amsalu ET, et al. Time to initiation of antenatal care and its predictors among pregnant women in Ethiopia: Cox-gamma shared frailty model. PloS One. 2021;16(2):e0246349. doi: 10.1371/journal.pone.0246349 33544714 PMC7864666

[20] Kuuire VZ, Kangmennaang J, Atuoye KN, Antabe R, Boamah SA, Vercillo S, et al. Timing and utilisation of antenatal care service in Nigeria and Malawi. Global Public Health. 2017;12(6):711–27. doi: 10.1080/17441692.2017.1316413 28441926

[21] Mbuagbaw L, Medley N, Darzi AJ, Richardson M, Garga KH, Ongolo‐Zogo P. Health system and community level interventions for improving antenatal care coverage and health outcomes. Cochrane Database of Systematic Reviews. 2015(12). doi: 10.1002/14651858.CD010994.pub2 26621223 PMC4676908

[22] Morestin F, Ridde V. The abolition of user fees for health services in Africa Lessons from the literature. Montreal: University of Montreal. 2009.

[23] McKinnon B, Harper S, Kaufman JS, Bergevin Y. Removing user fees for facility-based delivery services: a difference-in-differences evaluation from ten sub-Saharan African countries. Health policy and planning. 2015;30(4):432–41. doi: 10.1093/heapol/czu027 24816570 PMC4385820

[24] Sinyange N, Sitali L, Jacobs C, Musonda P, Michelo C. Factors associated with late antenatal care booking: population based observations from the 2007 Zambia demographic and health survey. The Pan African Medical Journal. 2016;25. doi: 10.11604/pamj.2016.25.109.6873 28292072 PMC5325499

[25] Gupta S, Yamada G, Mpembeni R, Frumence G, Callaghan-Koru JA, Stevenson R, et al. Factors associated with four or more antenatal care visits and its decline among pregnant women in Tanzania between 1999 and 2010. PloS one. 2014;9(7):e101893. doi: 10.1371/journal.pone.0101893 25036291 PMC4103803

[26] Ahinkorah BO, Seidu A-A, Budu E, Mohammed A, Adu C, Agbaglo E, et al. Factors associated with the number and timing of antenatal care visits among married women in Cameroon: evidence from the 2018 Cameroon Demographic and Health Survey. Journal of Biosocial Science. 2021:1–11. doi: 10.1017/S0021932021000079 33632369

[27] Bbaale E. Factors influencing timing and frequency of antenatal care in Uganda. The Australasian medical journal. 2011;4(8):431. doi: 10.4066/AMJ.2011.729 23393530 PMC3562883

[28] Finlayson K, Downe S. Why do women not use antenatal services in low-and middle-income countries? A meta-synthesis of qualitative studies. PLoS Medicine. 2013;10(1):e1001373. doi: 10.1371/journal.pmed.1001373 23349622 PMC3551970

[29] Pell C, Meñaca A, Were F, Afrah NA, Chatio S, Manda-Taylor L, et al. Factors affecting antenatal care attendance: results from qualitative studies in Ghana, Kenya and Malawi. PloS One. 2013;8(1):e53747. doi: 10.1371/journal.pone.0053747 23335973 PMC3546008

[30] Phillips E, Stoltzfus RJ, Michaud L, Pierre GLF, Vermeylen F, Pelletier D. Do mobile clinics provide high-quality antenatal care? A comparison of care delivery, knowledge outcomes and perception of quality of care between fixed and mobile clinics in central Haiti. BMC Pregnancy and Childbirth. 2017;17(1):1–11. 29037190 10.1186/s12884-017-1546-7 PMC5644158

