# Supplementary material for: Trends and determinants of late antenatal care initiation in three East African countries, 2007–2016: A population based cross-sectional analysis
Source: PLOS Glob Public Health. 2022 Aug 15;2(8):e0000534. doi: 10.1371/journal.pgph.0000534 (PMC10021240; doi:10.1371/journal.pgph.0000534)
Supplement: S1 Table — (DOCX) [file pgph.0000534.s001.docx]

**S1 Table: DHS survey data used in the study analysis.**

| **Country** | **Survey year** | **Survey type** | **Clusters** | **Sample** |
| --- | --- | --- | --- | --- |
| DRC |  |  |  |  |
|  | 2007 | Standard DHS-IV | 290 | 4, 559 |
|  | 2013/14 | Standard DHS-IV | 492 | 8,941 |
| Kenya |  |  |  |  |
|  | 2008 | Standard DHS-IV | 386 | 3,512 |
|  | 2014 | Standard DHS-IV | 1,573 | 13,776 |
| Tanzania |  |  |  |  |
|  | 2010 | Standard DHS-IV | 456 | 5,058 |
|  | 2015/16 | Standard DHS-IV | 608 | 6,873 |
| Combined Countries | 2007-2016 | Standard DHS | 3,805 | 42,719 |
